# Can the study of self-assembly in solution lead to a good model for the nucleation pathway? The case of tolfenamic acid.†
†Electronic supplementary information (ESI) available. See DOI: 10.1039/c5sc00522a


**DOI:** 10.1039/c5sc00522a

**Published:** 2015-04-17

**Authors:** W. Du, A. J. Cruz-Cabeza, S. Woutersen, R. J. Davey, Q. Yin

**Affiliations:** a School of Chemical Engineering and Technology , State Key Laboratory of Chemical Engineering , Tianjin University , Tianjin 300072 , People's Republic of China; b Van't Hoff Institute for Molecular Sciences , University of Amsterdam , Science Park 904 , 1098 XH Amsterdam , The Netherlands; c The School of Chemical Engineering and Analytical Sciences , The University of Manchester , Manchester M13 9PL , UK . Email: roger.davey@manchester.ac.uk

## Abstract

To further our understanding of the role of solution chemistry in directing nucleation processes new experimental and computational data are presented on the solution and crystallisation chemistry of tolfenamic acid (TA), a benchmark polymorphic compound. With these, and previously published data, we were able to establish that TA is rapidly fluctuating between conformers in solution with either solvated monomers or dimers present depending on the solvent. Hence, despite the fact that conformational polymorphs can be obtained from crystallisations in ethanol, we found no links between solution chemistry and crystallisation outcomes. We discuss the implications of these conclusions for the nature of the nucleation pathway *via* dimers and clusters and raise experimental questions about how best to undertake relevant crystallisation studies.

## Abstract 2

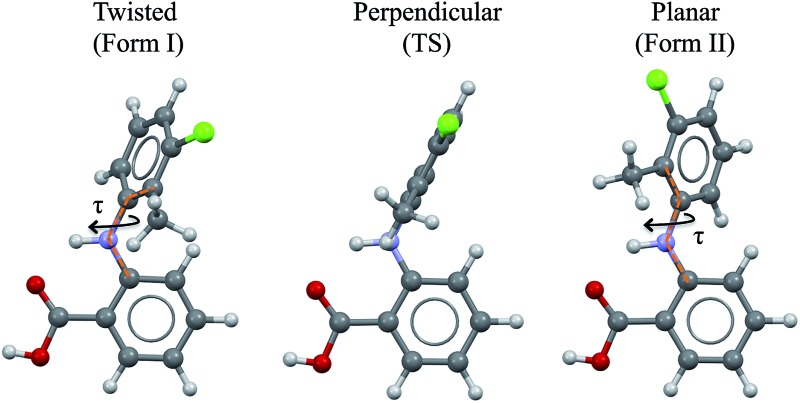
To further our understanding of the role of solution chemistry in directing nucleation processes new experimental and computational data are presented on the solution and crystallisation chemistry of tolfenamic acid (TA), a benchmark polymorphic compound.

## Introduction

In our quest to understand the molecular pathways involved in the nucleation of organic crystals from solutions, significant progress has been made over the last decade.^1^ One particular line of enquiry has sought to ask whether an enhanced appreciation of the nature and extent of molecular self-assembly in the solution phase can inform us further as to the changes in molecular association, molecular conformation and state of solvation occurring along the nucleation reaction co-ordinate. The early work in this field^2^–^5^ sought to exploit a combination of polymorphism, appropriate spectroscopies and computation, to examine the links between the so called ‘growth units’ present in solution and structural synthons,^6^ the supramolecular motifs which appear in the resulting crystal structures. This work led to the so-called ‘link’ hypothesis^7^ which is based on the idea that the structural outcomes of a crystallisation process reflect exactly the packing of the available clusters present at nucleation. The results of these studies have been reviewed recently by Davey *et al.*^1^ and it appears that there are indeed many cases in which spectroscopy reveals solution phase dimers which reflect rather well the synthons of the resulting crystal structures. It is clear that in such examples an inversion centre observed in a macroscopic crystal may have its origins in a dimerisation process taking place in solution. There are also examples in which the molecular conformation observed in the crystal structure is close to the expected solution phase conformer,^8^,^9^ again offering a link between solution chemistry and crystallisation outcome. Conversely, other materials (*e.g. R*,*S* mandelic and benzoic acids crystallised from alcohols) yield harvested crystals which contain centres of symmetry despite the fact that strong solvation prevents the formation of dimers in the solution-phase.^4^,^7^ Additionally there are examples (*e.g.* ethenzamide)^10^ in which the conformation appearing in the crystal is not an expected solution phase conformer. In such cases, an understanding of the solution chemistry offers no insight into where or how the essential features of the crystal structure might evolve.

In this contribution we explore further this central question of how much can really be learnt about nucleation from solution-phase experiments and associated computational studies. In particular we consider the essential levels of theory needed in the computational elements of such work; we question how best to characterise the solution species present; we also reflect on the question of how to measure the crystallisation outcomes of experiments so as to shed light on nucleation.

**Fig. 1:**
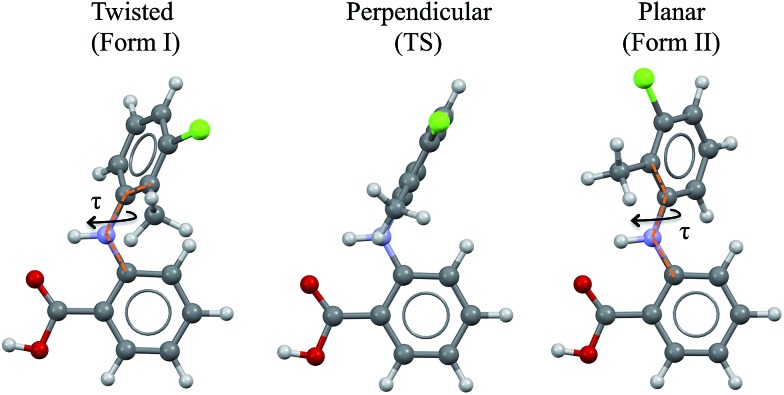
*Twisted*-like and *planar*-like conformations found in *Forms* I and II TA. The transition state (TS) between the observed conformations is also shown. The dotted orange lines define the torsion angle *τ* which is representative of the conformational change.

As a vehicle for this journey we utilise tolfenamic acid (TA), 2-[(3-chloro-2-methylphenyl) amino] benzoic acid (Fig. 1 and Table 1). This is a nonsteroidal anti-inflammatory drug with five structurally characterised polymorphs (CSD refcodes KAXXAI, KAXXAI01 – 04)^11^,^12^ and is an attractive model since it has been the subject of significant previous study. The most commonly encountered of the polymorphs, Forms I and II were first characterised by Andersen *et al.*^11^ These polymorphs are conformational because they contain two different gas-phase conformers:^8^ one twisted-like (found in Form I, referred to as *twisted* or *T* hereafter) and a second more planar-like (found in Form II, referred to as *planar* or *P* hereafter). These molecular conformations differ, as seen in Fig. 1, in the dihedral angle between the two phenyl rings, being ±73° in Form I (the white form) and ±46° in Form II (the yellow form). This conformational difference may also be expressed as the torsion angle about the nitrogen – phenyl carbon bond (*τ*) which is ±74.95° in Form I and ±142.63° in Form II. Although TA does not contain a chiral centre, its conformers are chiral. Because of the presence of an inversion centre in both polymorphs each crystal structure comprises both enantiomers (hence the ± sign for *τ*). The enantiomers can in principle interconvert by rotation about *τ*, but it is unlikely that this occurs spontaneously due to the high energy barrier. Further to this, it is also apparent that the transfer of the acid proton between oxygen atoms would offer the possibility of the existence of two tautomeric forms. Whilst both tautomeric forms have been studied in this work, the data for the metastable tautomer *B* is given in the ESI† only. The tautomer *A*, found in the solid state, is always the most stable in crystal, liquid and gas phases.

**Table 1:** Characteristic information on Forms I & II TA

	Form I	Form II
Refcode	KAXXAI01	KAXXAI
Space group	*P*2_1_/*c*	*P*2_1_/*n*
*Z*′	1	1
Colour	White	Yellow
Density exp. (110 K)	1.443	1.454
Conformation	Twisted-like (*T*)	Planar-like (*P*)
Angle between phenyl rings	±73°	±46°
*τ*	±75°	±143°

From earlier studies,^11^–^14^ there appears to have been some disagreement in the reported stability and thermodynamic relationship between Forms I and II TA. Our own results in this contribution agree with previous observations of an enantiotropic relationship, with Form I being the thermodynamically stable form at and above room temperature.

Mattei and Li have made an intensive study of the solution chemistry and crystallisation behaviour of TA Forms I and II.^14^ They recorded the polymorphic outcome of crystallisation experiments from ethanolic solution at 37 °C and found that with rising supersaturation, in the range 1.55 to 1.95, the stable Form I was increasingly favoured over the metastable Form II. Thus, at the lower supersaturations the system followed Ostwald's Rule with the initial appearance of the metastable Form II followed by its conversion to Form I. At the highest supersaturation only Form I, the thermodynamically stable form appeared. This is an unusual result and these authors sought an explanation through studies of the solution chemistry. UV/vis spectroscopy^14^ and NMR^15^ provided values of the self-association constants for TA in ethanol of *ca.* 140 M^–1^ and 31 M^–1^ at 25 °C by the two techniques. These were interpreted in terms of the existence of carboxylic acid hydrogen-bonded dimers (HBD) in the ethanolic solutions. The reported relationship between supersaturation and the relative appearance of the two forms was then rationalised through the idea that increasing solute concentration creates increasing numbers of dimers and that, based on computational results, the most stable dimer is that in which the molecules adopt the Form I conformation (twisted). Thus the rationale was that at low supersaturations the solutions are rich in monomers displaying the conformer of Form II (*planar*) whilst at high supersaturations the solutions are rich in dimers displaying the conformer of Form I (*twisted*). Nucleation was then assumed to dominate the crystallisation outcome and the observations related directly to the solution chemistry.

In our exploration of the solution chemistry – nucleation relations in this system we were first interested in re-examining the conformational energy relationships at varying levels of theory for monomer and dimers in both vacuum and solvent environments. Secondly, we wanted to review and extend the available spectroscopy in order to be clear about the nature of the solution species. Finally we wanted to extend the crystallisation experiments of Mattei and Li^14^ and to re-examine the notion of ‘crystallisation outcome’.

## Experimental section

### Computation of molecular geometries and energies

Conformer energies were computed in the gas-phase and with various implicit solvation models using GAUSSIAN09.^16^ Molecular models of tautomer A in planar and twisted geometries were retrieved from the experimental crystal structures whilst those of tautomer B were generated manually. The various molecular models were geometry optimised using tight convergence criteria at various levels of theory. Different DFT functionals, van der Waals corrections and basis sets were tested and are compared in the ESI.† The best compromise between computation time and accuracy for the calculation of conformer energies was found with the model comprising B97D/6-31+G(d,p).^17^ Tests on the accuracy of this model are presented in the ESI.† Geometry optimisations and the computed energetics at the B97D/6-31+G(d,p) level agreed extremely well with those computed with a double hybrid functional and a larger basis sets (B2PLYD/def2QVZPP). Geometry optimisations were performed in the gas-phase as well as in various solvents using the SMD implicit solvation models of Truhlar *et al.*^18^ Such SMD calculations were performed in six solvents namely toluene, ethylacetate, 2-propanol, ethanol, DMSO and water. For the calculation of geometries and energy for the conformations at the transition state between the planar and twisted minima, we used the Synchronous Transit-Guided Quasi-Newton 2 method^19^ as implemented in GAUSSIAN09.

### Computation of the potential energy surface for TA about *τ*

The potential energy surface (PES) of TA about *τ* was generated by optimising molecular geometries with *τ* constrained every 10° between –220° to 220°. All calculations were performed in an SMD solvation model for ethanol at the B97D/6-31+G(d,p) level of theory.

### Computation of dimer geometries and energies

Geometry optimisations and frequency calculations of several dimer and monomer models were computed, free of constraints, at the B97D/6-31+G(d,p) level of theory in the gas-phase and in the six different SMD solvent models. The Gibbs free energy (*G*) is the sum of the electronic energy plus the thermal free energy (*G*(*T*) = *E*_e_ + *G*_corr_(*T*) where *G*_corr_(*T*) is calculated from the frequency analysis). *E*_e_ was re-computed *via* a single point energy calculation of the optimised geometries with the same functional but a larger basis set (B97D/def2QZVPP). The use of the large basis sets for this calculation ensures minimisation of the basis set superposition error. To minimise computational costs, the *G*_corr_ term was computed with the smaller basis set B97D/6-31+G(d,p) model only. The free energies of the dimers were then calculated at different temperatures as the difference between the free energy of the dimer (with either planar or twisted conformation) minus the free energy of two monomers with the planar conformation (since this is the most stable conformer according to our models).§
§Δ*G*_d_(*T*) = *G*_dimer-planar/twisted_(*T*) – *G*_mono-planar_(*T*) – G_mono-planar_(*T*). Similar models have recently been used for the computational study of self-association of various carboxylic acids in solution.^20^

### NMR calculations

NMR chemical shifts were computed using the Gauge-Independent Atomic Orbital method^21^ as implemented in GAUSSIAN09. The NMR calculations were performed on the B97D/6-31+G(d,p) optimised monomer and dimer geometries at the same level of theory. The chemical shifts in the text are reported relative to those of tetramethylsilane (TMS) calculated in the same way.

### Materials and analytical tools

Tolfenamic acid Form I (CAS no. 13710195, >98% purity) was purchased from Sigma Aldrich and used without further purification. TA Form II was crystallised by crash cooling an ethyl acetate solution (3.45 g TA Form I and 50.00 g of ethyl acetate) to 10 °C. Both forms were isolated as pure phases as judged by their powder X-ray diffraction (PXRD) patterns. Ethyl acetate (EtOAc), ethanol and 2-propanol were purchased from VWR International Ltd. (UK), toluene from Fischer Chemicals and deuterated ethanol (EtOD) from Sigma Aldrich (>99.5%D). All solvents were of analytical reagent grade and the molar purities were >99.5%.

Powder X-ray diffraction (PXRD) was performed using a Rigaku miniflex X-ray powder diffractometer at a wavelength of 1.5406 Å controlled by DIFFRACPLUS software from 4° to 40° with a step size of 0.03°.

The FTIR spectra of solutions of TA in EtOD and deuterated toluene were recorded in 0.50 or 1.00 mm thick liquid-sample cells, using a Perkin Spectrum Two spectrometer with 2 cm^–1^ resolution. The spectra were corrected for the (small) solvent contribution by recording solvent spectra in the same liquid cell and subtracting these from the solution spectra.

Differential Scanning Calorimetry (DSC) experiments were performed using either a Mettler Toledo DSC 30 instrument controlled by Mettler TC15 complete with a liquid nitrogen cooling system with data analyzed by STARe software v.610 or a TA DSC Q100 with software universal analysis 2000 v. 4.5A. A heating rate of 10 K min^–1^ was used.

### Crystallisation experiments

The crystallisation of TA was investigated in crash cooling experiments in toluene, ethylacetate, 2-propanol and ethanol. These experiments were carried out using a 50 mL jacketed vessel with an overhead 2-blade impeller stirring at 200 rpm. Solutions at different concentrations were prepared by dissolving the corresponding amount of TA Form I in 40 g solvent. The solutions were kept at 60 °C for 1 h to ensure that all the crystals were dissolved completely. 10 mL aliquots of the solutions were then withdrawn and filtered through a pre-heated 0.2 μm syringe filter, transferred to the jacketed vessel pre-set to the desired crystallisation temperature (Thermo Scientific DC10, UK). The crystals were filtered immediately after nucleation and dried at room temperature for 0.5 h. Each experiment was repeated 5 times and both PXRD and visual observation (colour) were used to identify the polymorphic forms of the product crystals.

## Results

### Stability and solubility of the forms

Lattice energy calculations, thermal analysis, slurry and solubility measurements were used to determine the thermodynamic relationship between Forms I and II TA (see ESI†). Forms I and II are related enantiotropically with a transition temperature below 0 °C. Form II is the low temperature form whilst Form I is the most stable form in the temperature range studied here. From the ratio of solubilities in 2-propanol, the free-energy difference between the two forms was calculated to be 0.3 kJ mol^–1^ at 10 °C.

### Relative stability of conformers and the transition state

The relative stability of the TA conformers and the energy barriers for conformational change are key for the understanding of the role played by conformational flexibility during crystallisation. We have computed the stability of the various possible monomeric species of TA using several computational methods and compared the results with previous literature reports in the ESI.† It is evident that the conformational energies computed for this system are sensitive to the theoretical models used. This has recently been discussed for fenamate-type molecules by Price *et al.*^22^Table 2 contains a summary of the relative stability of the conformers and transition state of TA in the tautomeric form A and in different solvation media with the B97D/6-31+G(d,p) model. The relative stability of the twisted and planar conformers was found to change slightly with solvent and the energy barrier for their interconversion is typically ∼5 kJmol^–1^ (or just over 1 kcal mol^–1^).

**Table 2:** Relative stability of conformers and the transition state (*TS*)^*a*^ of TA tautomer A in various media of dielectric constant *ε* at 0 K

Medium	*ε*	Relative conformational energies (kJ mol^–1^)		
		*Twisted*	*TS*	*Planar*
Gas-phase	1	–0.2	4.4	0.0
Toluene	2	1.1	5.4	0.0
EthylAcetate	6	0.8	5.4	0.0
2-Propanol	19	0.3	5.2	0.0
Ethanol	25	0.3	5.2	0.0
DMSO	47	0.5	5.6	0.0
Water	78	–0.3	4.2	0.0

^*a*^Relative energy and geometry optimisations at the B97D/6-31+G(d,p) level of theory.

**Fig. 2:**
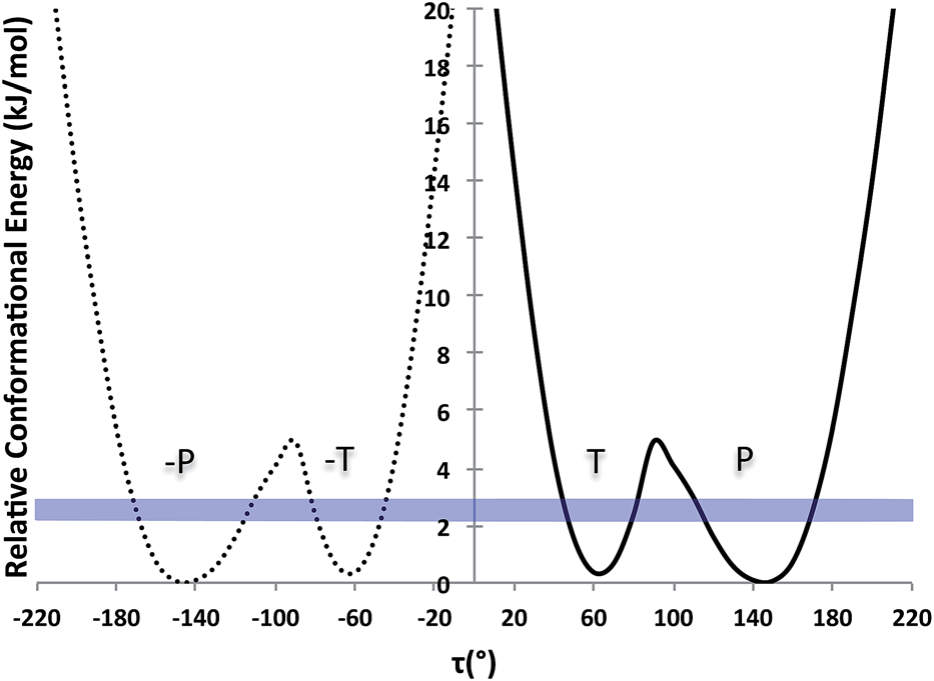
PES scan of TA tautomer A as a function of *τ* in ethanol (*τ* step = 10°). Two curves are plotted (one per enantiomer) and RT at the crystallisation conditions is given as a blue band.

Further, we computed the potential energy surface (PES) of TA about the rotatable bond *τ* in ethanol also for tautomer A and for both enantiomers (Fig. 2). The energy barrier to go from one enantiomer to the other *via* rotation about *τ* was calculated to be around 25 kJ mol^–1^. This is very high and so unlikely to occur at ambient conditions. The energy barrier to go from the T to the P conformer for a given enantiomer, however, is around 5 kJ mol^–1^, just above thermal energy. Hence, for a given enantiomer, interconversion between the P and the T conformers should be facile.

### Possible modes of self-assembly

**Fig. 3:**
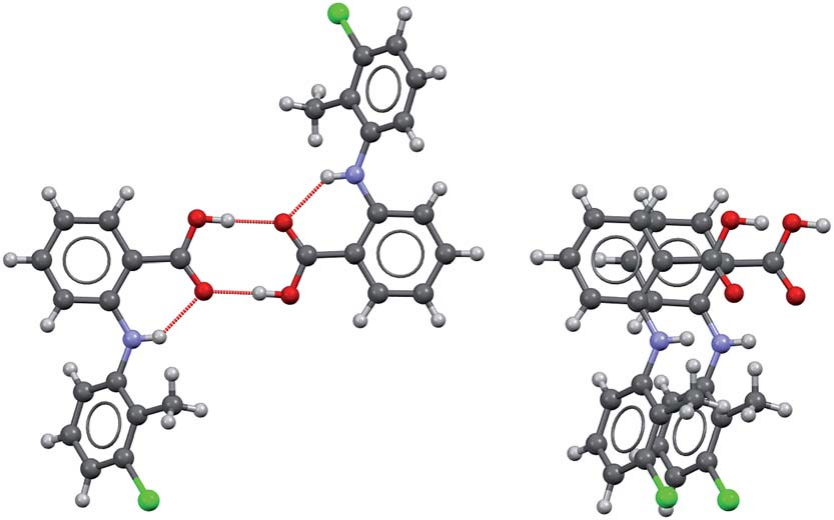
Hydrogen-bonded (left) and stacked (right) dimers of TA.

Considering the crystal structures of Forms I and II TA as guides, two types of self-assembly modes may be envisaged for TA (Fig. 3): (1) through hydrogen bonding (hydrogen-bonded dimers, HBD) or (2) through aromatic stacking (stacked dimers, SD). We note that HB dimerisation may occur between opposite or identical enantiomers. Since we expect HBDs between opposite and identical enantiomers to be energetically similar, we have only considered those related by inversion because they are the ones observed in the crystal structures. Dimerisation *via* stacking as presented in Fig. 3, however, can only occur between two molecules in the same enantiomeric form. We have computed dimerization energies (0 K) and free energies (at three temperatures) for HBDs and SDs built from twisted and planar conformers in various solvents (Table 3).

**Table 3:** Dimerisation energies (Δ*E*_d_) and free energies (Δ*G*_d_) for TA tautomer A as HBDs and SDs in various media of dielectric constant *ε*

	*ε*	Type^*a*^	Δ*E*_d_ (kJ mol^–1^)	Δ*G*_d_ (kJ mol^–1^)		
			0 K	283 K	298 K	310 K
Gas-phase	1	HBD-T	–63	–13	–10	–8
		HBD-P	–66	–19	–17	–15
		SD-T	–54	10	13	15
		SD-P	–58	4	7	9
Toluene	2	HBD-T	–52	–4	–1	1
		HBD-P	–57	–5	–3	–1
		SD-T	–36	25	28	31
		SD-P	–42	18	20	23
EtOAc	6	HBD-T	–48	11	14	16
		HBD-P	–53	1	3	5
		SD-T	–39	22	25	27
		SD-P	–46	21	24	26
2-Propanol	19	HBD-T	–35	20	22	25
		HBD-P	–38	13	15	17
		SD-T	–47	17	20	22
		SD-P	–56	9	12	14
Ethanol	25	HBD-T	–34	19	22	24
		HBD-P	–38	14	16	18
		SD-T	–48	16	19	21
		SD-P	–57	7	10	12
DMSO	47	HBD-T	–49	11	14	17
		HBD-P	–50	2	5	7
		SD-T	–48	15	18	20
		SD-P	–56	8	11	14
Water	78	HBD-T	–38	12	15	17
		HBD-P	–41	13	16	18
		SD-T	–60	–2	1	3
		SD-P	–70	–7	–4	–2

^*a*^Type of dimer and conformation. *T* for *twisted* and *P* for *planar*.

It is clear that (Table 3) solvation of a TA molecule/dimer is very different in solvents of different type with dimerisation energies depending significantly on solvent. Negative dimerisation energies and free energies indicate that the dimer configuration is more stable than two independent monomers in that particular medium. We observe that at temperatures other than 0 K most monomers are favoured over dimers. This is due to the entropic penalty associated with dimerisation at higher temperatures. At temperatures other than 0 K the only exceptions in which dimers are favoured over monomers are: (1) HBDs in the gas phase, (2) HBDs in toluene and (3) SDs in water. In ethanol solutions, we observed that dimer formation is particularly disfavoured.

In comparing the relative free energies of HBDs to SDs at room temperature, it appears that HBDs are always preferred in non-polar media (gas, toluene, EtOAc) while both types of dimer are similarly stable in alcohols and DMSO. Only in water do SDs become more stable than HBDs. With respect to the molecular conformation, dimers with the planar conformer usually appear to be more stable than those with the twisted one. The energy differences, however, are small so dimerization is unlikely to be a cause of conformational restriction.

### What species exist in ethanol solutions?

**Fig. 4:**
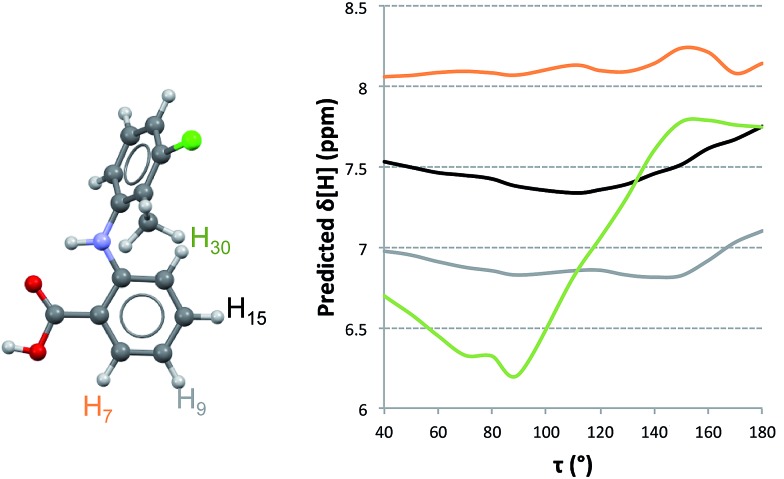
Calculated changes in chemical shifts with torsion angle *τ* for four selected hydrogen atoms. H30 is the aromatic hydrogen atom displaying the most significant changes in chemical shift with molecular conformation.

Proton NMR experiments of TA solutions have been performed by Andersen *et al.*^11^ and, more extensively, by Mattei *et al.*^15^ In the former ^1^H-NMR spectra of TA in acetone were recorded at various temperatures. They concluded that the spectra did not show significant variations in the range 210–290 K, indicating that TA does not exist in any favoured conformation under those conditions. Mattei *et al.*^15^ studied the variation of ^1^H-NMR chemical shifts in ethanol with temperature and concentration and observed only very small variations in chemical shifts. This was particularly true as a function of concentration (typically of the order of 0.001 ppm) where the observed dependencies were interpreted in terms of molecular dimerisation in solution at increasing concentrations and decreasing temperatures. It was explicitly assumed that this dimer was identical to the H-bonded dimer found in the crystal structures and, following their computational results, that such a solution dimer should bear the twisted conformation. We have reexamined the available experimental data^15^ in an attempt to correlate the measurements with NMR predictions of chemical shifts for TA. In particular, we examined the experimental dataset measured on 1.5 mM ethanolic solutions of TA at 25 °C (because lower concentration solutions should have a dominance of monomer species) and the data measured at the highest concentration, 62.5 mM ethanolic solutions of TA at 25 °C. Our computed results are shown in Fig. 4 and Table 4 where, for consistency and ease of comparison with previously reported data, we use the original atom labelling (H7, H9, H15 and H30).^15^

**Table 4:** Experimental and predicted chemical shifts for protons H7, H9, H15 and H30. R^2^ and a and b values derived from linear regression between the experimental and predicted values (*δ*_exp_ = *aδ*_pred_ + *b*) are also given

Chemical Shifts [ppm]											
	Experimental^*a*^		Computed Mono		Average Mono	Computed HBD		Average HBD	Computed SD		Average SD
	[1.5 mM] at 298 K	[62.5 mM] at 298 K	Mono-T	Mono-P		HBD-T	HBD-P		SD-T^*b*^	SD-P^*b*^	
H7	7.98	7.98	8.09	8.20	8.14	8.12	8.19	8.15	7.58	7.61	7.59
H9	6.71	6.70	6.90	6.81	6.86	6.89	6.98	6.97	6.61	6.47	6.54
H15	7.27	7.26	7.46	7.48	7.47	7.41	7.43	7.42	7.02	7.03	7.02
H30	6.83	6.82	6.41	7.71	7.06	6.38	7.84	7.11	6.09	7.55	6.82
*R* ^2^	—	—	0.852	0.655	**0.996**	0.858	0.544	**0.994**	0.811	0.342	**0.968**
*a*	—	—	0.732	0.805	**1.014**	0.721	0.818	**1.097**	0.826	0.637	**1.282**
*b*			1.914	1.120	**–0.292**	1.996	0.969	**–0.941**	1.548	2.623	**–1.777**

^*a*^Experimental chemical shifts were retrieved from the H-NMR spectra as given in ref. 23 (Fig. 3.2).

^*b*^Computed by averaging the chemical shifts over the calculated ones for the two independent molecules of the dimer.

To understand how the chemical shifts of the different protons change with conformation, we computed NMR chemical shifts for all conformations studied in the PES of TA at values of *τ* in 10° increments. It is clear from Fig. 4 that the chemical shift of H30 varies more significantly with changes in conformation than those for the other three hydrogen atoms. We also see that the relative ordering of chemical shifts changes with variations in conformation. Experimentally, the ordering of chemical shifts (lower to higher) is H9 < H30 < H15 < H7. We note that, according to our predictions (Fig. 4), this experimental ordering of chemical shifts is only achieved in a small range of *τ* values between 110° and 135°.

In order to compare how well the chemical shift predictions for the two conformers of TA reproduce the experimental values, we carried out linear regression on the predicted against the experimental chemical shifts (Table 4). NMR chemical shifts were computed for each conformer and fitted to the experimentally reported values using an equation of the type *δ*_exp_ = *aδ*_pred_ + *b*. This procedure was done twice, first with the monomeric species (fitting the predicted chemical shifts to the experimental values derived from the lowest concentrated solution) and second with the dimer species (fitting the predicted chemical shifts to the experimental values derived from the highest concentrated solution).

The resulting values for *a* and the good of fitness *R*^2^ are presented in Table 4. It is evident that predictions with either the twisted or the planar conformers (in monomer or dimer aggregates) do not reproduce the measured chemical shifts. When an average chemical shift is calculated, however, the fits between the predicted and experimental values are excellent for the monomer and HBDs and worse for the SDs. This suggests that both conformers are in equilibrium and since they interconvert very quickly, we only observe a single signal per proton which corresponds to an average NMR value. Hence, based on the NMR data together with the NMR predictions, it must be concluded that in ethanol TA is fluctuating very fast around *τ* and that there is no preferred conformation.

With respect to aggregation, the experimental H-NMR chemical shifts of low and high concentrated solutions hardly change (see ref. 23 and Table 4). The calculations, however, in going from the average monomer predictions to the average stacked dimer predictions, show a considerable decrease in chemical shifts (of a few ppms). We conclude from these data that stacked dimers should not exist in solution. In going from the average monomer predictions to the average HB dimer predictions, however, there is only a slight increase in the predicted chemical shifts. With such small differences in chemical shifts between the monomers and the HBDs, proton NMR would not be able to discriminate between monomers and HB dimers.

**Fig. 5:**
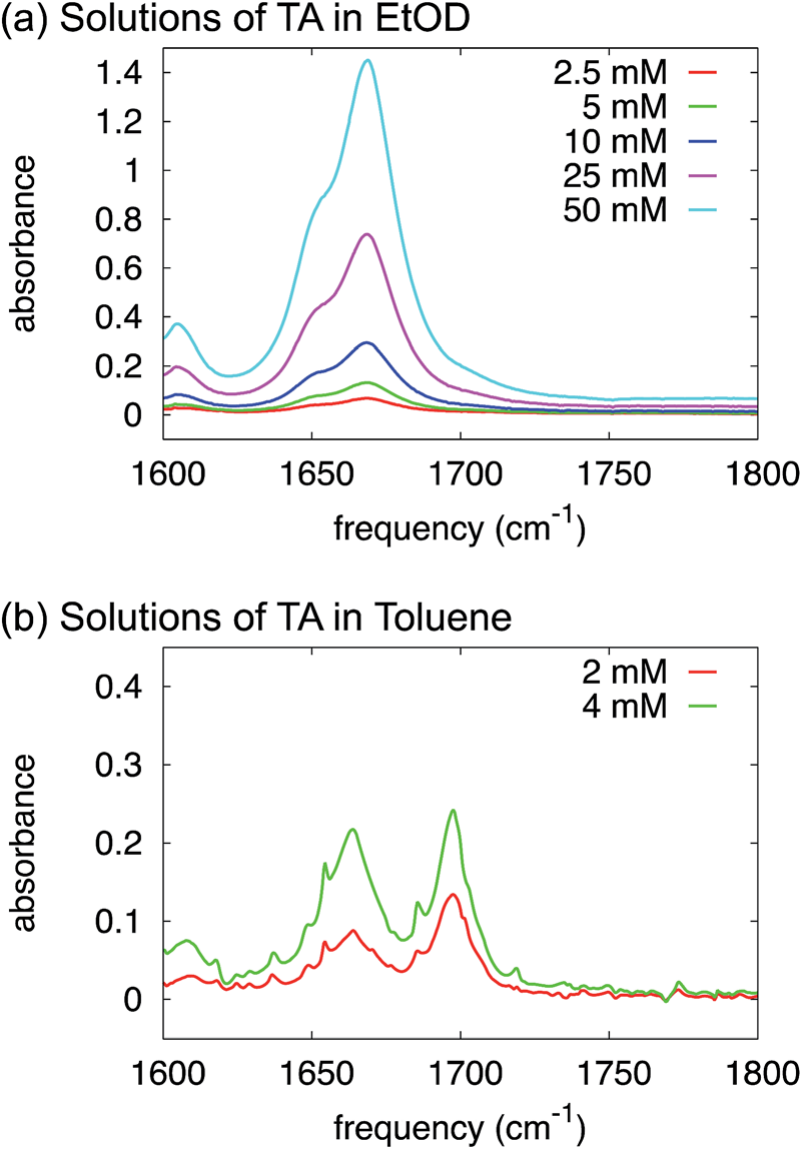
FTIR spectra of solutions of TA in deuterated ethanol (a) and toluene (b) at varying concentrations.

In order to shed further light on this issue we have used FTIR to monitor the band associated with stretching of the C=O group (∼1700 cm^–1^) of TA in ethanol as a function solution concentration. The C=O-stretch mode is a very sensitive probe of carboxylic-acid dimerization. This is partly because of hydrogen-bond induced weakening of the C=O bond, but mainly because transition-dipole coupling between the two C=O bonds in a dimer results in a Raman-active symmetric and an IR-active antisymmetric C=O-stretch mode which both have frequencies very different from that of the monomer.^24^ Typically, the IR-active C=O-stretch of a dimer has a frequency 40–50 cm^–1^ lower than the monomeric C=O-stretch (the precise value depends on the solvent).^25^ Hence, in a mixture of monomers and dimers, two peaks are visible (the monomer C=O-stretch and the dimer antisymmetric C=O-stretch), and their relative intensities depend strongly on concentration: at low concentration the monomer peak dominates, at high concentration the dimer peak. In the IR spectra of TA in ethanol (Fig. 5a) we observe two C=O-stretch peaks, but the frequency splitting is only 20 cm^–1^, much less than would be expected for a monomer-dimer difference. More importantly, the intensity ratio of the two peaks is completely independent of concentration, which we varied over more than an order of magnitude (Fig. 5a). Hence, the presence of two C=O-stretch peaks cannot be due to TA dimerization. We ascribe this difference to a TA : ethanol hydrogen-bonding equilibrium (the low- and high-frequency C=O-stretch frequencies corresponding to hydrogen-bonded and non-hydrogen-bonded CO groups, respectively). In fact, a solution of *N*-methylacetamide in methanol has a similar, concentration-independent two-peak C=O-stretch spectrum due to the formation of C=O···H–O hydrogen bonds by part of the molecules,^26^ the frequency splitting being 20 cm^–1^, exactly the same value as observed here.

Our overall conclusion from both the existing NMR data and new FTIR results is that ethanolic solutions of TA are unlikely to contain hydrogen-bonded or stacked dimers, rather the solutions are populated with solvated monomeric species in which the chlorine-containing ring oscillates between the twisted and the planar conformers. The calculations carried out in the previous section also suggest that monomers are preferred over dimers in ethanol solutions.

### What species exist in toluene solutions?

We have proven in the previous sections that TA exists as a monomer in ethanol solutions. But, what species should be favoured for TA in toluene? Intuitively, toluene is a non-polar aromatic solvent so we might expect it to interact more strongly with the aromatic side of the TA molecule but not with the carboxylic acid group. In fact, the calculations in Table 3 suggest that HB dimers of TA are thermodynamically favoured in toluene.

In order to test this experimentally, just as in the section above, we have used FTIR to study the variations in relative intensity of the C=O band for TA solutions of various concentrations in toluene (Fig. 5b). In contrast to ethanol, we can clearly identify monomeric and dimeric C=O-stretching peaks at ∼1700 cm^–1^ and ∼1660 cm^–1^ respectively. The intensity of the dimer band at ∼1660 cm^–1^ increases relative to the intensity of the monomer band at ∼1700 cm^–1^ in going from a 2 mM to a 4 mM solution of TA in deuterated toluene (Fig. 5b). The frequency difference for these bands is about 40 cm^–1^, which is close to the typical value observed for carboxylic-acid monomers-dimers equilibria.^25^ We note that the narrow peaks superimposed on the C=O-stretch spectrum are due to water-vapour absorption (see ESI†). This experiment, therefore, clearly suggests that HB dimers are present in solutions of TA in toluene.

### Crystallisation outcomes

**Fig. 6:**
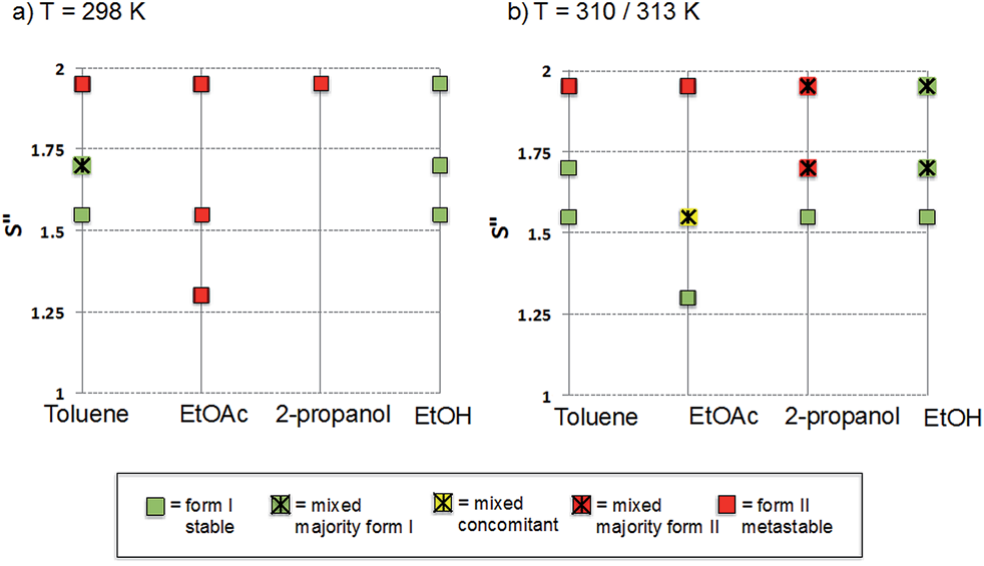
Crystallisation outcomes as a function of supersaturation and solvent at 298 and 310/313 K. Pure green and red squares correspond to experiments for which at least four out of the five crystallisations performed resulted in pure Forms I and II respectively. Crossed squares represent mixed outcomes with a majority (at least three out of five crystallisations) of form I (green), II (red) or both (yellow, concomitant).

The polymorphic outcomes of crash cooling crystallisation in toluene, ethyl acetate, 2-propanol and ethanol at different temperatures (*T*) and supersaturations (*S*) were studied. Detailed results are given in the ESI† whilst we present the crystallisation results for two sets of temperatures (25 and 37 °C/40 °C) in Fig. 6. Precipitation (induction) times were observed to be between seconds to minutes and dependent on supersaturation, solvent and temperature. Evidently, at a given temperature, increasing supersaturation results in shorter induction times. Transformation times, however, between the forms are of the order of hours suggesting that the forms arise from direct precipitation and not *via* transformation.

The data of Fig. 6 show two major features. Firstly increasing temperature appears to yield more products which appear as mixtures of forms. This is not unexpected given the activated nature of the crystallisation process. From the perspective of the current study however, the major observation is that independent of solvent, higher supersaturations favour the appearance of the metastable Form II while lower supersaturations favour the appearance of the stable Form I. This is the expected behavior in a polymorphic system and essentially follows Ostwald's Rule of Stages.

Given the discussion above concerning the solvated nature of TA in various solvents, it is expected that in changing from the least polar (toluene) to the most polar solvent (ethanol) solutions will go from being dimer to monomer rich. Using this insight, together with the data in Fig. 6, it can be concluded that the dimer/monomer state of TA in solution does not affect the overall polymorphic outcome. Both polymorphs contain the HB dimers and it is now clear from these new computations and crystallisation results that solution phase dimerization does not lead to a “lock” in conformation as has been postulated before.^14^ If this were the case then dimer rich toluene solutions would yield only form I crystals: this is clearly not so. In a sense, and contrary to previous conclusions, there is nothing out of the ordinary that requires explanation and this is consistent with the notion expressed here that there are no favoured conformations in solution, irrespective of the existence of dimers. If one wished to make a link between solution chemistry and crystallisation, then it would have to explain more generally why in this and many other polymorphic systems the metastable form is favoured under conditions of high supersaturation.

## Discussion

Firstly it seems clear from both the computations and our comments on the available NMR data that for TA, conformation change is facile so that a crystal nucleating or growing from a monomer rich solution will be insensitive to the existence of the two conformers. Hence each molecule is a potential growth unit and we would expect no conformationally driven outcomes when crystallisation occurs from monomeric solutions. If crystallisation takes place from a dimer-rich solution then the situation is unchanged since the computations suggest that H-bonded dimers (like monomers) show no significant conformational preference. Our analysis shows no experimental evidence from NMR to support the existence of a preferred conformation in ethanol solutions.

Considering dimerization, on the one hand ethanol solutions appear to favour the solvation of the acid group and are hence, monomer rich. Toluene solutions, on the other hand, seem to favour the formation of HB dimers. Crystallisation results, however, show that both monomer and HB dimer containing solutions follow Ostwald's Rule of Stages.

In considering the creation of crystal nuclei it is worth noting two points. Firstly there is nothing about the solution chemistry that would select a particular conformer for incorporation into a critical cluster. Secondly, while the existence of solution phase hydrogen-bonded dimers between enantiomer pairs could offer a pathway for the creation of the required crystallographic inversion centre, their absence means that both the conformation and the centre of symmetry must be determined at some other stage during nucleation. How this occurs remains an open question. For example, the expulsion of solvent from a disordered aggregate might enable the formation of dimers: with HB dimers a molecule has only to find its mirror image to begin the process of crystallisation. However, if we make an analogy with micellisation, a clustering phenomenon of amphiphiles in polar solvents, then we might imagine that a critical assembly of TA molecules has an aromatic core with acid groups at its surface, making use of the solvating power of the solvent. In this situation the drive towards crystalline order may be initiated by the attainment of stacking interactions. However since stacked dimers as found in Forms I & II comprise only one enantiomer, the centre of symmetry must be created through a subsequent self-assembly process between stacks and this might be seen as less likely. The development of H-bonded dimers and consequent centres of symmetry (which are present in all known and also in all putative polymorphs)^27^ would thus require a second, as yet unvisualised but thermodynamically driven step.

Finally, we note that other type of aromatic interactions between two TA molecules can be generated by inversion and are observed in Forms III and IV. However, these are less energetically favoured than the SDs considered here as found in Forms I & II. Interestingly, Forms III & IV can only be nucleated on surfaces of non-polar aromatic polymers.^12^

## Conclusions

In attempting to understand the relationship between crystal nucleation, solution chemistry and molecular conformation, it may be considered that TA is a suitable benchmark system. Prior to our current work, existing publications suggested that all that should be done on this system had been done – the crystal structures of two conformationally distinct forms had been reported, phase behavior and solubilities had been measured, crystallisation outcomes were recorded under well-defined conditions and significant investigations of solution chemistry made. All this had been combined with computational chemistry to develop a self-consistent interpretation relating crystallisation to dimerisation and molecular conformation.

Having repeated and extended both experimental and computational aspects of this system, it now appears that there is no link between its solution chemistry and conformational polymorphism. Both additional experiments and higher-level computations have shed new light on the energetics of TA conformers and on the species existing in ethanolic solutions. It now appears that a comprehensive nucleation model for this system must be able to explain how the conformational polymorphs arise from a solution in which there is *no* energetic preference for the solid state conformers and in which there are no dimers to offer a pathway for symmetry and structural control during the self-assembly process. Importantly, it is worth considering how to proceed both experimentally and computationally when exploring such problems and what data we lack in trying to resolve the molecular issues surrounding the nucleation transition state.

The initial impetus for study of this system came from the apparently anomalous outcome of the original crystallisation experiments which we were unable to repeat. Crystallisation is a response to an intimate combination of phase equilibria and kinetics and so we are not surprised that different workers, in different labs, using different equipment, chemicals of different purity and ethanol of different water contents should obtain different results. This possibility is well known to those active in the field and reflected in so-called ‘disappearing polymorphs’.^28^

Perhaps a more important consideration is the question of exactly which crystallisation experiments should be pursued to define the nucleation characteristics of a given system. Workers in the field have become used to the idea that structural characterisation of the final macroscopic crystals in any given experiment provides a link to nucleation. In their 1983 paper concerning the crystallisation of stearic acid polymorphs from cyclohexanone, Sato and Boistelle^29^ were careful to interpret their measured occurrence domains in terms of both relative growth and nucleation rates of the three forms. Around the same time Cardew and Davey^30^–^32^ showed that in a dimorphic system it is the relative magnitude of the product of the nucleation rates, *J* and the growth rate constants, *k*^3^, of the two polymorphs that determines the experimental outcome. Subsequent workers, however, have continued to use the final, macroscopic outcome of crystallisation experiments to infer structural information about nucleation, ignoring the contribution made by growth.^33^ More recently workers^34^ have returned to the idea that the specific measurement of nucleation rates might be a better way forward and indeed the use of induction time distributions to collect these data shows considerable promise.^35^

The computational results of different workers should not be subject to the same variation in outcomes as the experiments. Our work confirms this but additionally highlights the need to perform calculations at suitable levels of theory in order to achieve the most reliable results, at least for this molecule in which intra- and intermolecular interactions need to be accurately modeled.

Finally we note the difficulty in characterizing uniquely the state of molecular assembly in solutions. Solutions and their dynamic nature are of course fully characterised only in terms of the radial distribution functions describing the environment of the different atoms on the solute/solvent pair. Techniques such as NMR will only be useful in cases where dimers or conformers are particularly stable and abundant. FTIR offers a different view on the state of interactions of specific functionalities with their environment but as yet from the position and intensity of a particular absorbance it is hard to make a unique assignment concerning the intermolecular interactions involved. Certainly, to assume *a priori* that a motif present in a crystal structure is also present in solution is a very dangerous assumption to make.

## Supplementary Material

Supplementary information
