# Supplementary material for: Can the study of self-assembly in solution lead to a good model for the nucleation pathway? The case of tolfenamic acid
Source: Chem Sci. 2015 Apr 17;6(6):3515–24. doi: 10.1039/c5sc00522a (PMC5814770; doi:10.1039/c5sc00522a)
Supplement: Supplementary file 1 [file SC-006-C5SC00522A-s001.pdf]

Electronic Supplementary Material (ESI) for Chemical Science.
This journal is © The Royal Society of Chemistry 2015

SUPPLEMENTARY INFORMATION:
Can the study of self-assembly in solution lead to a good model for the nucleation pathway? The case of tolfenamic acid.

A. J. Cruz-Cabeza[a], W. Du[b], R. J. Davey[c]*, S. Woutersen[b] and Q. Yin[a]

S1. Contents

This ESI contains detailed information on the data presented or referred to in the main manuscript. Methods are described first (S2 experimental and S3 computational) followed by extensive explanations of the results (section S4).

S2. Experimental Methods

S2.1 Materials and Analytical Tools

Tolfenamic acid Form I (CAS no.13710195, >98% purity) was purchased from Sigma Aldrich and used without further purification. TA Form II was crystallised by crash cooling an ethyl acetate solution (3.45 g TA Form I and 50.00 g of ethyl acetate) to 283.15 K. Both forms were isolated as pure phases as judged by their powder X-ray diffraction (PXRD) patterns. Ethyl acetate (EtOAc), ethanol and 2-propanol were purchased from VWR International Ltd. (UK) and deuterated ethanol (EtOD) from Sigma Aldrich (>99.5%D). All solvents were of analytical reagent grade and the molar purities were > 99.5%.

Powder X-ray diffraction (PXRD) was performed using a Rigaku miniflex X-ray powder diffractometer at a wavelength of 1.5406 Å controlled by DIFFRACPLUS software from 4° to 40° with a step size of 0.03°.

The FTIR spectra of solutions of TA in EtOD were recorded in 0.50 or 1.00 mm thick liquid-sample cells, using a Perkin SpectrumTwo spectrometer with 2 cm^{-1} resolution. The spectra were corrected for the (small) solvent contribution by recording solvent spectra in the same liquid cell and subtracting these from the solution spectra.

Differential Scanning Calorimetry (DSC) experiments were performed using either a Mettler Toledo DSC 30 instrument controlled by Mettler TC15 complete with a liquid nitrogen cooling system with data analyzed by STARe software v.610 or a TA DSC Q100 with software universal analysis 2000 v. 4.5A. A heating rate of 10 $Kmin^{-1}$ was used.

S2.2 Crystallisation Experiments

The crystallisation of TA was investigated in crash cooling experiments in toluene, ethyl acetate, 2-propanol and ethanol. These experiments were carried out using a 50 mL jacketed vessel with an overhead 2-blade impeller stirring at 200 rpm. Solutions at different concentrations were prepared by dissolving the corresponding amount of TA Form I in 40g solvent. The solutions were kept at about 333.2 K for 1 h to ensure that all the crystals were dissolved completely. 10 mL aliquots of the solutions were then withdrawn and filtered through a pre-heated 0.2 μm syringe filter, transferred to the jacketed vessel pre-set to the desired crystallisation temperature (Thermo Scientific DC10, UK). The crystals were filtered immediately after nucleation and dried at room temperature for 0.5 h. Each experiment was repeated 5 times and both PXRD and visual observation (colour) were used to identify the polymorphic forms of the product crystals.

S2.3 Solubility Measurements

The solubilities of TA Form I in toluene, ethyl acetate, 2-propanol and ethanol were measured as functions of temperature in the range of 283.2 – 333.2 K. Excess amounts of pure Form I were added to 20 mL of solvent to saturate the solutions and create a slurry. The desired temperature was maintained by a thermostatic water bath (Thermo Scientific DC10, UK) with an accuracy of ±0.1 K. After being stirred for 24 h at each temperature, the suspension was allowed to settle for 30 min. Then a 5 mL sample of the clear solution was taken using a syringe with a membrane filter (0.20 μm). The residue of undissolved crystals was separated and identified to be the starting polymorph by PXRD, indicating that no solvent-mediated polymorphic transformation occurred during solubility measurement experiments. Samples of the saturated solution were dried at room temperature until the solvent was completely evaporated. The solubility was determined from the mass of the remaining crystalline material and the total solution.

The solubility of TA Form II was measured only in 2-propanol. Experiments were performed in a 15 mL jacketed vessel agitated with a magnetic stirrer. Form II prepared as described above was stirred with 2-propanol for 1 h in the temperature range of 283.2 -333.2 K. Liquid samples were withdrawn through a pre-warmed 0.2 μm syringe filter and analysed for TA content using UV absorption at 343 nm after dilution. Five samples at certain concentrations were prepared to obtain a set of standards for the construction of a standard curve for quantitative concentration analysis.

S2.4 Solution-mediated Polymorphic Transformation

The solution-mediated polymorphic transformation between Forms I and II was investigated in ethyl acetate at 273.2, 278.2, 280.2 and 283.2 K, respectively. 0.20 g of Form II, 0.02 g Form I of TA and 10 mL ethyl acetate were added into a jacketed vessel. This slurry was stirred with a magnetic stirrer and the temperature was controlled by a thermostat with anti-freezing liquids as the cooling material. The progress of the transformation was judged from colour variation of the two polymorphic forms and the crystals were filtered and dried at room temperature when the colour of the slurry turned into white or yellow. Finally the polymorphic form was confirmed by PXRD.

S3. Computational Methods

S3.1 Calculation of the forms relative lattice energies.

Crystallographic data of Forms I and II TA were retrieved from the CSD (refcodes KAXXAI01 and KAXXAI[1] respectively). The coordinates of the experimental crystal structures were used as input parameters for geometry optimisations with the program VASP.[2–5] The functional PBE[6] was used with PAW[7,8] pseudo potentials and Grimme's van der Waals corrections.[9] A kinetic energy cut-off of 520 eV was applied for the plane-waves. The Brillouin zone was sampled using the Monkhorst-Pack[10] approximation on a grid of k-points separated by around 0.05 Å during the optimisations. Structural relaxation was stopped when the calculated force on every atom of the cell was less than 0.003 eV/Å. After geometry relaxations, a single point energy calculation of the lattice was performed using a finer grid of k-points (separated by 0.01 Å).

S3.2 Computation of Molecular Geometries and Energies

Conformer energies were computed in the gas-phase and with various implicit solvation models using GAUSSIAN09.[11] Molecular models of tautomer A in planar and twisted geometries were retrieved from the experimental crystal structures whilst those of tautomer B were generated manually. The various molecular models were geometry optimised using tight convergence criteria at various levels of theory. Different DFT functionals, van der Waals corrections and basis sets were tested and are compared in section S4. We use the SMD implicit solvation models of Truhlar *et al.*[12] Such SMD calculations were performed in six solvents namely toluene, ethylacetate, 2-propanol, ethanol, DMSO and water. For the calculation of geometries and energy for the conformations at the transition state between the planar and twisted minima, we used the Synchronous Transit-Guided Quasi-Newton 2 method as implemented in GAUSSIAN09. [11]

S3.3 Computation of Dimer Geometries and Energies

Geometry optimisations and frequency calculations of several dimer and monomer models were computed, free of constraints, at the B97D/6-31+G(d,p) level of theory in the gas-phase and in the five different SMD solvent models. The Gibbs free energy (G) is the sum of the electronic energy plus the thermal free energy ($G(T) = E_e + G_{corr}(T)$, $G_{corr}(T)$ is calculated from the frequency analysis). E_e was computed via a single point energy calculation of the optimised geometries at the B97D/def2QZVPP model. The use of the large basis sets for this calculation ensures minimisation of the basis set superposition error. To minimise computational costs, the G_{corr} term was computed with the B97D/6-31+G(d,p) model. The free energies of the dimers were then calculated at different temperatures as the difference between the free energy of the dimer minus the free energy of the planar monomer ($\Delta G_{AB}(T) = G_{AB}(T) – G_A(T)– G_B(T)$).

S3.5 NMR Calculations

NMR chemical shifts were computed using the Gauge-Independent Atomic Orbital method[21] as implemented in GAUSSIAN09. The NMR calculations were performed on the B97D/6-31+G(d,p) optimised monomer and dimer geometries at the same level of theory. The reported chemical shifts in the text are calculated relative to the computed chemical shifts of tetramethylsilane (TMS) -using the same computational models.

S4. Results

S4.1 Identification of Forms I and Form II

The experimental PXRD patterns of the unground pure white and yellow crystals obtained in these experiments are compared with the calculated patterns in Figure S1.

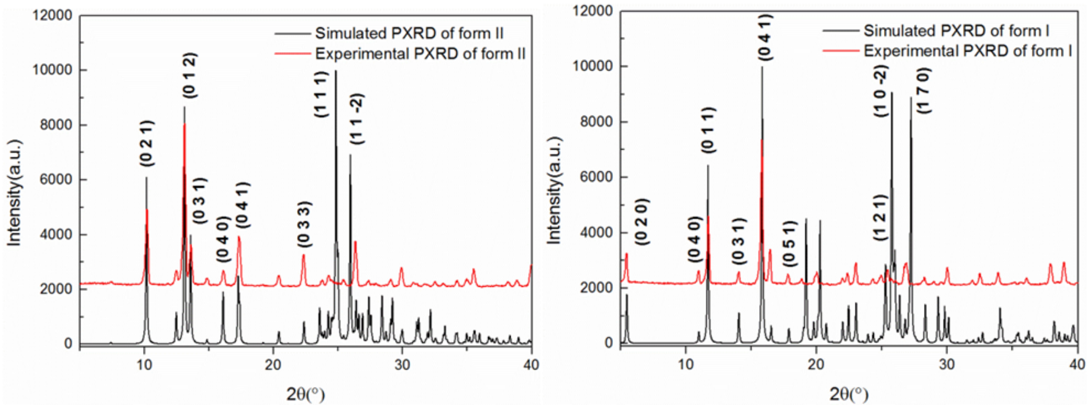

(a) (b)

Figure S1. Powder X-ray diffraction patterns of unground TA crystals compared to the calculated patterns: (a) Form I and KAXXAI01, (b) Form II and KAXXAI.

The PXRD comparisons confirm the samples to be pure Forms I and II respectively. It is noted that the intense peaks around 2θ = 25° in the calculated patterns are essentially missing from the experimental patterns. This is certainly due to preferred orientation since the crystals of both forms are known to be highly anisotropic a-axis needles. Thus the major peaks observed for Form I represent the reflections (0 2 0), (0 4 0), (0 1 1) and (0 3 1) and for Form II (0 2 1), (0 1 2), (0 3 1) and (0 4 1), confirming that both forms are indeed elongated in the direction of the a-axis.

Beyond the PXRD patterns, the colour of the forms are quite different and indicative of their structure. Form I is white whilst Form II is yellow.

S4.2 Solubility of Forms I and II in various solvents

The measured solubilities of Form I in ethanol, 2-propanol and ethyl acetate and that of II in 2-propanol are shown in Figure S2. Additionally, numerical data for the solubilities in toluene are given in table S1. We notice that TA is considerably less soluble in toluene than in other tested solvents.

The data for Form I are compared to its ideal solubility calculated using the approximate relationship (ignores the heat capacity correction) equation 1:

$$\ln x_{eq} = \frac{\Delta H_f}{R}\left[\frac{1}{T_f} - \frac{1}{T}\right] \qquad (1)$$

where x_{eq} is the mole solubility, ΔH_f is the heat of fusion (34.51 kJ·mol^{-1}), T_f is the melting temperature (484.2 K) and T is the absolute temperature. It can be seen from Figure S2 that the ideal solubility curve essentially divides the data into two groups: ethyl acetate having solubility higher than the ideal curve and ethanol and 2-propanol with solubilities lower than the ideal. It can also be seen from Figure S2 that the solubilities of Form I and Form II in 2-propanol are very close with the solubility of Form II consistently higher than that of Form I in the tested temperature range. This confirms the fact that Form I is the more stable form over the studied temperature range. The ratio of solubilities ($RT\ln X_{II}/X_I$) of the two forms in

2-propanol at 10 °C gives a value of 0.31 kJmol^{-1} for the free energy difference between the two forms.

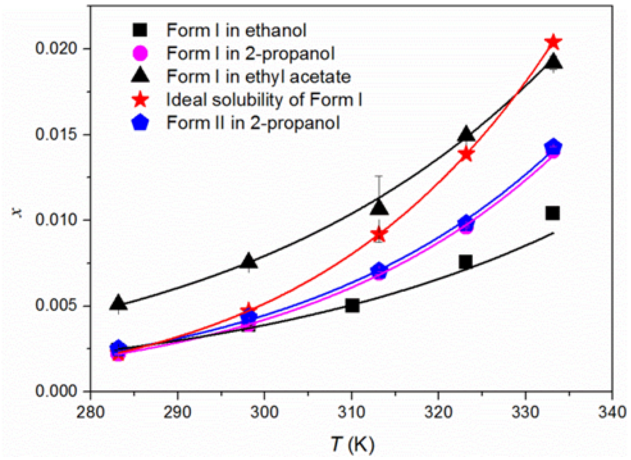

Figure S2. Temperature dependence of the solubility of TA Form I in ethyl acetate (▲), as calculated ideally (★), 2-propanol (●) and ethanol(■) and of TA Form II in 2-propanol (⬟).

Table S1. Solubility measurements for TA Form I in toluene.

Solvent	T	C* (g/100g solvent)	Average value (g/100g solvent)	*x* (mole fraction)
Toluene	283.15	0.1273	0.1274	0.0004
		0.1275		
		0.0458		
	298.15	0.1967	0.1928	0.0007
		0.1988		
		0.1829		
	313.15	0.4602	0.4430	0.0016
		0.4591		
		0.4096		

S4.3 The thermodynamic relationship between Forms I and II.

As briefly noted in the main article, there appears to be some disagreement about the thermodynamic relationship between forms I and II TA. In order to clarify this our own thermal analysis and theoretical calculations are compared here with the previously reported data.

Thermal analysis led Andersen *et al*[1] to suggest that the forms are enantiotropically related with Form II the stable low temperature modification and Form I the stable form above ~ 0°C (the transition temperature was calculated to lie around 273.15-283.15 K). Surov *et al*[13] also found Form II to be the low temperature form via solution calorimetry; however, they concluded that the transition temperature of the forms is above their melting temperatures (the transition temperature was calculated to lie around 493.8 K) and hence, Form II is always the most stable up until melting and the polymorphs are related monotropically. By contrast, Lopez-Mejias et al[14] reported the relative free energies of all polymorphs as determined by conversion and optical absorbance experiments at room temperature. They found form I as being the thermodynamically favoured form at room

temperature. Mattei and Li[15] also demonstrated, from a combination of solubility and thermal analysis, that Form I is the more stable of the polymorphs at room temperature and above. They did not comment on the stability of the forms at low temperature. These authors also recorded an endothermic event in the DSC of form II at 141 ^{o}C which they ascribed to a solid-solid form II to form I transformation (DSC event which was not seen by Lopez-Mejias[14] or Andersen[1]).

In DSC experiments, we also observed an endothermic peak at 157 ^{o}C when heating Form II which is the form II to form I solid-solid phase transition. We note that Mattei and Li's lower temperature for the transition (141 ^{o}C)[15] reflects both the kinetic nature of this phenomenon with different samples behaving slightly differently and the potential for sublimation of samples during heating. According to the heat of transition rule,[16] the existence of a solid-solid phase transition below the melting point suggests the polymorphs have an enantiotropic relationship. From our solution-mediated transformation experiments in ethyl acetate it was hoped to identify the transition temperature. However, even at 0 ^{o}C Form II was found to transform to Form I. Transformation times were not recorded in detail – at the higher temperatures (10 ^{o}C) transformations started at 16.00 hrs were complete overnight while at 0 ^{o}C they took noticeably longer. Our calculations of the relative energies of Form I and Form II at 0 K (Table S2) indicate that Form II has the lowest energy and hence, also support the conclusion that Form II is the more stable polymorph at lower temperatures. The relative stabilities of the forms appear to be very close to the enthalpy differences reported by both Andersen *et al*[1] and Surov *et al*[13] but differ significantly in magnitude from the endothermic event recorded in the DSC (corresponding to the solid-solid transformation) both in this work and by Mattei and Li.[15]

Table S2. Recorded thermal events, thermodynamic relationship and calculated relative energy of Forms I and II TA from various independent studies.

	**Form I**	**Form II**	**Stable Low T**	**Stable Room T**	**Relationship**
**Previous Studies**					
ΔH (kJ/mol) - Andersen 1989*	5.9	0	Form II	Form I	Enantiotropic
ΔH (kJ/mol) - Surov 2009†	6.7±1.2	0	Form II	Form II	Monotropic
ΔG (kJ/mol, 300K) – Lopez-Mejias 2009**	0	0.2	-	Form I	-
$\Delta H_{solid\text{-}solid}$ (kJ/mol) - Mattei 2012 ‡	1.1	0	-	Form I	-
**This study**					
$\Delta H_{solid\text{-}solid}$ (kJ/mol) - Exp.‡	0.7	0	Form II	Form I	Enantiotropic
ΔG (kJ/mol, 285K) – Exp.***	0	0.3	Form II	Form I	Enantiotropic
ΔE (kJ/mol) - Calculated DFT-d	5.9	0	Form II	-	-

*From differences in heat of fusion, original paper by Andersen et al.[1]
†From solution calorimetry.[13]
** From conversion and optimcal absorbance experiments.[14]
‡ Solid-solid transformation of form II into form I (DSC).[15]
*** From solubility ratio at 10 °C.

In summary, the majority of data indicate that Forms I and II of TA are enantiotropically related with a transition temperature around or below 0 ^{o}C (or 273.15 K). Form II is the most

stable form at low temperatures and Form I is the thermodynamically stable form above ~0 °C. Entropic contributions, hence, must be more important in the free energy of Form I at higher temperatures. Andersen *et al*[1] in fact already noticed this in their comment: "as judging from the thermal ellipsoids, thermal and entropic effects do play a more important role in form I than in form II".

S4.4 Choice of Computational Model

We have evaluated the conformational energies of the twisted and planar conformers of TA, as well as the transition state (Figure 1) in the gas-phase, using various DFT models and basis sets. Our results are given together with previously reported values in table S3. We have used a meta-GGA (M06-2X),[17] a GGA with dispersion (B97-D)[9] and a double hybrid with dispersion (B2PLYP-D). The relative stability of the twisted conformer and the TS are both given respect to the planar conformer (table S3). There are some variations in absolute numbers with changes in basis-sets and DFT functional, however, all methods predict both conformers to be similarly stable (almost isoenergetic with some models). As we can see from table S3, the energetics predicted with our best model (B2PLYPD/def2QZVPP) are reproduced very well by the much less computationally expensive B97D/6-31G+(d, p). We have chosen, therefore, to use this model for the calculation of conformer energies through this work.

Table S3. Stability of conformers of TA tautomer A in the gas-phase as calculated with various computational methods.

	Conformational Energy Relative		
**Study and Method**	**Twisted**	**TS***	**Planar**
**Mattei and Li**[18]			
B3LYP/6-31G(d,p)//MP2/6-31+G(d,p)	~4	-	0.0
**Uzoh et al.**[19]			
PBE0/6-31+G(d,p)	1.5	2.9 (2.9)	0.0
HF/6-31G(d,p)//MP2/6-31+G(d, p)	1.8	4.8 (4.8)	0.0
**This study**			
B97D/6-31+G(d,p)	-0.2	4.6 (4.8)	0.0
B97D/aug-cc-pVTZ	0.8	6.1 (6.1)	0.0
B97D/def2QZVPP	0.8	6.1 (6.1)	0.0
M062x/6-31+G(d,p)	-2.5	1.7 (4.2)	0.0
M062x/aug-cc-pVTZ	-1.2	3.3 (4.5)	0.0
M062x/def2QZVPP	-1.2	3.9 (5.1)	0.0
B2PLYPD/6-31+G(d,p)	-1.7	2.6 (4.3)	0.0
B2PLYPD/ def2QZVPP §	-0.3	4.3 (4.6)	0.0

*Relative energy of the TS respect to the planar conformer and relative energy of the TS respect to the lowest energy conformer in parenthesis.

§ Single point energy calculation on the geometry optimised at the B2PLYPD/6-31+G(d,p) level of theory.

S4.5 Stability of Tautomers and Conformers in Various Solvents

As dicussed in the introduction of the manuscript, TA can adopt two tautomeric forms: A and B (Figure S3). We evaluated the relative stability of these at the B97D/6-31+G(d,p) level of theory in various solvents making use of the SMD solvation models. The results are summarised in Table S4.

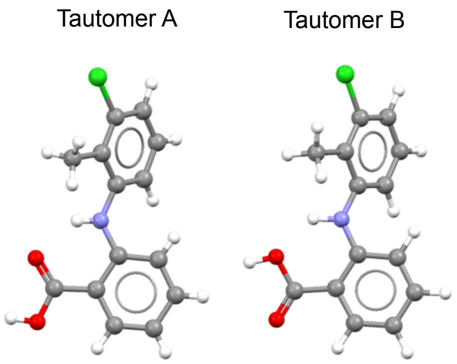

Figure S3. Tautomers A and B in TA. Only tautomer A is observed in the solid state.

Table S4. Relative stability of tautomers, conformers and transition states of TA in various media. Geometry optimisations were performed at the B97D/6-31+G(d,p) level of theory.

		Stability relative to Planar Tautomer A (kJ/mol)					
		Tautomer A			Tautomer B		
**Medium**	**ε**	**Twisted**	**TS**	**Planar**	**Twisted**	**TS**	**Planar**
Gas-Phase	1	-0.2	4.6	0.0	13.5	18.6	14.1
Toluene	2	1.1	5.4	0.0	13.0	17.5	12.2
EthylAcetate	6	0.8	5.4	0.0	11.3	16.1	10.8
2-Propanol	19	0.3	5.2	0.0	8.3	13.3	8.2
Ethanol	25	0.3	5.2	0.0	7.9	13.0	7.9
DMSO	47	0.5	5.6	0.0	9.9	15.2	9.6
Water	78	-0.3	4.2	0.0	6.6	11.9	7.2

The relative stability of the TA tautomers strongly depends on the solvent nature. For example, whilst the conformers of tautomer B are ~14 kJ/mol less stable than those of tautomer A in the gas phase, these energy differences are reduced to just ~ 7 kJ/mol in water. In all cases, however, tautomer A is more stable than tautomer B. We have, therefore, only considered tautomer A for the dimer calculations.

S4.6 Results from crystallization experiments

Crystallisation experiments were performed in various solvents at different temperatures and supersaturations. Every experiment for a given point in the phase space was

repeated five times (expect for S=1.95, 10 °C in toluene which was repeated four times). The polymorphic outcomes of the crystallisation experiments are summarised in Table S5.

Crystallisation results for 25 °C and 37/40 °C are also given in the main manuscript (Figure 6). If at least four out of five crystallisation experiments gave one form uniquely, a green (form I) or a red (form II) symbols were used in figure 6. For less than four equal crystallisation outcomes, the squares with crosses symbols were used.

The major observation from these crystallisation results is that higher supersaturations favour the appearance of the metastable Form II while lower supersaturations favour the appearance of the stable Form I.

Table S5. Summary of the polymorphic forms obtained from crash cooling crystallisations with various solvents, supersaturations (S) and temperatures. 5 independent experiments were repeated for each set of conditions.

Solvent	S	10			25 °C			37 °C			40 °C			60 °C		
		I	II	I+II	I	II	I+II	I	II	I+II	I	II	I+II	I	II	I+II
Toluene	1.55				4	1	0				4	1	0			
	1.70				3	2	0				5	0	0			
	1.95	3	1	0	0	5	0				1	4	0			
Ethyl acetate	1.30				0	5	0				4	0	1			
	1.55				0	5	0				0	0	5			
	1.95	0	5	0	0	5	0				0	4	1			
	3.00	0	5	0												
2-propanol	1.55										5	0	0	3	2	0
	1.70										0	2	3			
	1.95				0	5	0				0	3	2			
	3.00	4	0	1	0	1	4									
ethanol	1.55				5	0	0	4	1	0						
	1.70				4	0	1	3	0	2						
	1.95	5	0	0	5	0	0	3	0	2						
	3.00	0	0	5												
	5.00	2	0	3												

S4.7 FTIR of TA

We used FTIR to monitor the band associated with stretching of the C=O group (~ 1700 cm^{-1}) of TA in toluene and ethanol (see main article, Figure 5), as a function solution concentration. The C=O-stretching band is very sensitive to dimerization due to hydrogen-bond induced weakening of the C=O bond and to transition-dipole coupling between the C=O bonds.[20] As explained in the main text, the C=O-stretch peak of a dimer typically has a frequency 40–50 cm^{-1} lower than the monomeric C=O-stretch peak, and with increasing concentration, the dimer peak should increase in intensity relative to the monomer peak.[21] Whilst this behaviour is not observed for TA in ethanol (see Fig S5), it is clearly observed in toluene (Fig S4). This observation suggests that hydrogen-bonded TA dimers exist in toluene, but not in ethanol.

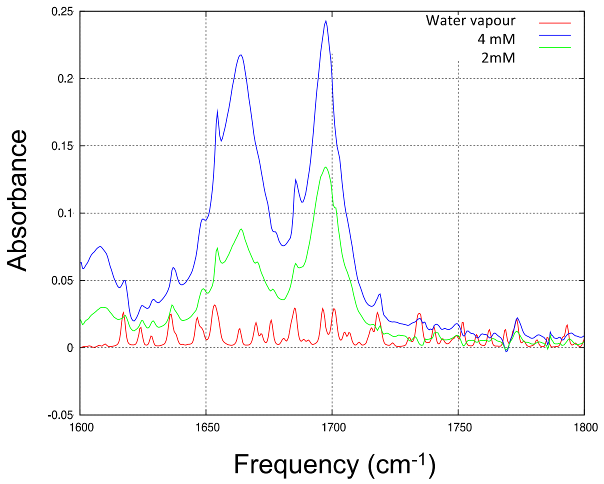

Figure S4. FTIR spectra of solutions of Tolfenamic acid in deuterated toluene at 2mM and 4mM concentrations. The solvent is substracted from the spectrum. The monomeric and dimeric C=O-stretch peaks are at ≈1660 and ≈1700 cm^{-1}. The narrow peaks superimposed on the C=O-stretch spectrum are due to water-vapour absorption (the spectrum of water vapour is shown for comparison as the grey curve).

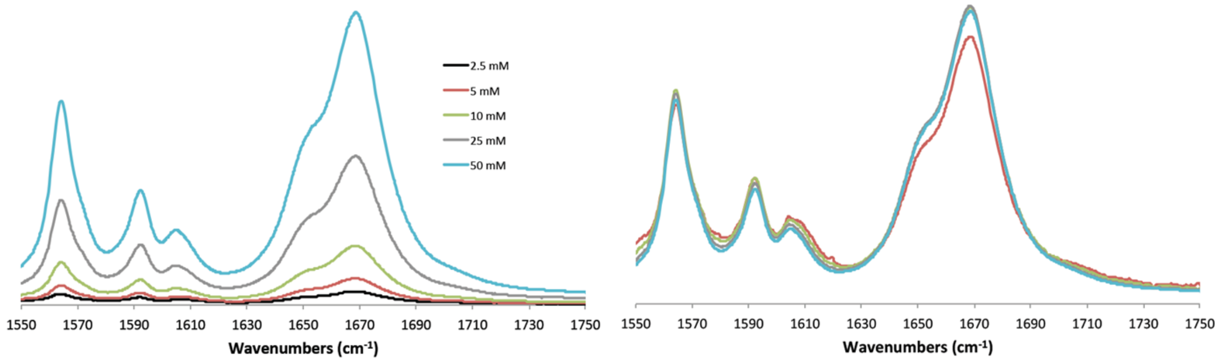

Figure S5. Unscaled (left) and scaled (right) FTIR spectra of solutions of Tolfenamic acid in deuterated ethanol at varying concentrations.

S5. References

1 K. V. Andersen, S. Larsen, B. Alhede, N. Gelting and O. Buchardt, *J. Chem. Soc. Perkin Trans. 2*, 1989, 1443–1447.
2 G. Kresse and J. Hafner, *Phys. Rev. B*, 1993, **47**, 558.
3 G. Kresse and J. Hafner, *Phys. Rev. B*, 1994, **49**, 14251.
4 G. Kresse and J. Furthmüller, *Comput. Mater. Sci.*, 1996, **6**, 15–50.
5 G. Kresse and J. Furthmüller, *Phys. Rev. B*, 1996, **54**, 11169.
6 J. P. Perdew, K. Burke and M. Ernzerhof, *Phys. Rev. Lett.*, 1996, **77**, 3865–3868.
7 P. E. Blöchl, *Phys. Rev. B*, 1994, **50**, 17953.
8 G. Kresse and D. Joubert, *Phys. Rev. B*, 1999, **59**, 1758.
9 S. Grimme, *J. Comput. Chem.*, 2006, **27**, 1787–1799.

10 H. J. Monkhorst and J. D. Pack, *Phys. Rev. B*, 1976, **13**, 5188–5192.
11 M. J. Frisch, G. W. Trucks, H. B. Schlegel, G. E. Scuseria, M. A. Robb, J. R. Cheeseman, G. Scalmani, V. Barone, B. Mennucci and G. A. Petersson, *GAUSSIAN 09*, Gaussian Inc., Wallingford, CT, 2009.
12 A. V. Marenich, C. J. Cramer and D. G. Truhlar, *J. Phys. Chem. B*, 2009, **113**, 6378–6396.
13 A. O. Surov, P. Szterner, W. Zielenkiewicz and G. L. Perlovich, *J. Pharm. Biomed. Anal.*, 2009, **50**, 831–840.
14 V. López-Mejías, J. W. Kampf and A. J. Matzger, *J. Am. Chem. Soc.*, 2012, **134**, 9872–9875.
15 A. Mattei and T. Li, *Pharm. Res.*, 2012, **29**, 460–470.
16 A. Burger and R. Ramberger, *Microchim. Acta*, 1979, **72**, 273–316.
17 Y. Zhao and D. G. Truhlar, *Theor. Chem. Acc.*, 2008, **120**, 215–241.
18 A. Mattei and T. Li, *Int. J. Pharm.*, 2011, **418**, 179–186.
19 O. G. Uzoh, A. J. Cruz-Cabeza and S. L. Price, *Cryst. Growth Des.*, 2012, **12**, 4230–4239.
20 J. Dybal, T. C. Cheam and S. Krimm, *J. Mol. Struct.*, 1987, **159**, 183–194.
21 Y. Fujii, H. Yamada and M. Mizuta, *J. Phys. Chem.*, 1988, **92**, 6768–6772.
